# Factors Influencing the Efficiency of Public Hospitals in Saudi Arabia: A Qualitative Study Exploring Stakeholders' Perspectives and Suggestions for Improvement

**DOI:** 10.3389/fpubh.2022.922597

**Published:** 2022-06-16

**Authors:** Ahmed D. Alatawi, Louis W. Niessen, Minakshi Bhardwaj, Yussif Alhassan, Jahangir A. M. Khan

**Affiliations:** ^1^Department of Clinical Pharmacy, College of Pharmacy, Jouf University, Sakaka, Saudi Arabia; ^2^Departments of International Health and Clinical Sciences, Liverpool School of Tropical Medicine, Liverpool, United Kingdom; ^3^College of Nursing, Midwifery and Health Care, University of West London, Middlesex, United Kingdom; ^4^Department of International Public Health, Liverpool School of Tropical Medicine, Liverpool, United Kingdom; ^5^Health Economics and Policy Unit, School of Public Health and Community Medicine, University of Gothenburg, Gothenburg, Sweden; ^6^Department of Learning, Informatics, Management and Ethics, Karolinska Institute, Stockholm, Sweden

**Keywords:** hospital efficiency, public hospitals, key informants interviews, health policy, qualitative analysis, Kingdom of Saudi Arabia

## Abstract

**Objective:**

Despite an extensive literature on efficiency, qualitative evidence on the drivers of hospital efficiency is scant. This study examined the factors that influence the efficiencies of health service provision in public hospitals in the Kingdom of Saudi Arabia (KSA) and their potential remedies.

**Design:**

We employed a qualitative design involving semi-structured interviews conducted between July and September 2019. Participants were purposively selected and included policymakers and hospital managers drawn from districts, regional and national levels. Data were analyzed in Nvivo 12 based on a thematic approach.

**Setting:**

Key informants of Ministry of health in the KSA.

**Results:**

Respondents identified a range of different factors across the community, facility and the wider health system that influence inefficiencies in public hospitals in KSA. Ineffective hospital management, lack of strategic planning and goals, weak administrative leadership, and absence of monitoring hospital performance was noted to have a profound impact on hospital efficiency. The conditions of healthcare staff in respect to both skills, authority and psychological factors were considered to influence the efficiency level. Further, lack of appropriate data for decision making due to the absence of an appropriate health informatics system was regarded as a factor of inefficiency. At the community level, respondents described inadequate information on the healthcare needs and expectations of patients and the wider community as significant barriers to the provision of efficient services. To improve hospital efficiencies, respondents recommended that service delivery decisions are informed by data on community health needs; capacity strengthening and effective supervision of hospital staff; and judicious resource allocation.

**Conclusion:**

The study demonstrates that inefficiencies in health services remain a critical challenge in public hospitals in KSA. Extensive awareness-raising and training on efficient resource utilization among key health systems stakeholders are imperative to improving hospital performance. More research is needed to strengthen knowledge on hospital efficiency in light of the limited data on the topic in KSA and the wider Gulf region.

## Strengths and Limitations of This Study

- The study reviewed components, mechanisms, barriers, and remedial actions of efficiency in public hospitals from the viewpoints of MOH policymakers and healthcare professionals.
- This study is the first qualitative research in identifying possible influential factors and recommendations for hospital efficiency in Saudi Arabia.
- The relatively small sample size (20 participants) might be a limitation of the study. However, we employed purposive and snowball sampling to identify participants with valuable insights needed to address the study objectives. We also kept the saturation of information under consideration in our analyses.
- The key informants were from three different levels of the healthcare system, which enabled to capture of a wide range of viewpoints to understand the factors of inefficiency and create feasible action-plans that based on real-life experience.

## Introduction

During the recent decades, the Kingdom of Saudi Arabia (KSA) has experienced a substantial increase in population growth, life expectancy, high prevalence of lifestyle diseases like cardiovascular diseases and incidences of infectious diseases, which increased the demand for health services and health spending (1–3). The public expenditure on healthcare was 67.8% of the country's total health expenditure, corresponding to 3.9% of GDP for 2016 (4). Such spending has dramatically increased by 24.7% from 2013 to 2017 (5). However, the healthcare statistics and studies conducted in KSA showed considerably lower availability of services, given the high health expenditure in KSA compared with other high-income countries, which indicated inefficiency in health service delivery (4, 6).

The rising healthcare expenditures and growing demand for healthcare services have placed the obligation for developing an effective, equitable and efficient healthcare system in most nations worldwide, including KSA (7, 8). The World Health Report in 2010 has estimated that about 20–40% of all healthcare spending (between $1.3–$2.6 trillion) is wasted globally due to inefficiency in the healthcare system (9, 10). Moreover, such loss of healthcare resources due to hospital-related inefficiency was valued at $300 billion annually (9). Since hospitals are the main consumers of healthcare resources, their efficiency is crucial to the overall efficiency of healthcare systems (6, 7, 11–14). Therefore, governments are required to conduct an efficiency analysis of their healthcare sectors and identify the causes of inefficiency to undertake necessary policies and practices for ensuring the effective utilization of public resources (6, 15). It is essential to determine inefficiency in healthcare systems and understand the factors that affect the efficiency of public hospitals (6).

The government of KSA guarantees free access to medical care for all citizens in public sector's facilities throughout the country based on article 31 of the national constitution (16). The Ministry of Health (MOH) is the primary provider of healthcare services in KSA, which administers 60% of all healthcare providers through public facilities and secures 75% of total healthcare expenditures in the country (17). Additionally, the MOH plans healthcare strategies, formulates healthcare policies, supervises healthcare service delivery programs, health education and monitoring of all health-related activities in KSA (18, 19). MOH provides primary, secondary and tertiary healthcare services through 2,361 primary healthcare centers and 282 public hospitals, including 43,080 beds in 2018 (5, 19).

The concept of technical efficiency suggests a hospital produces the maximum amount of outputs from a given input or a given output with the least quantities of inputs (20, 21). Research on the technical efficiency assessment of public hospitals and the factors that affect efficiency is rarely conducted in KSA. A systematic review and meta-analysis on public hospital efficiency studies in the Gulf region have shown the number of studies limited in findings (6). The review found only two studies conducted in KSA. The first one was done in 2017, which assessed the efficiency of healthcare services at a district level in KSA during 2014. The average technical efficiency score was 0.92; also, 45% of the districts achieved the efficient score (22). Another efficiency analysis was conducted in 2013 using data from 20 public hospitals under private sector management, which found that 60% of the study sample had not achieved the efficient score (23). Our efficiency analysis of 91 public hospitals affiliated to MOH was conducted (24). The study found that most public hospitals (75.8%) were technically inefficient, with the wasted amount of one-quarter of the total health resources used in the hospitals (24).

The literature emphasized that the performance of hospitals is influenced by internal and external factors (e.g., environmental factors) that are beyond the control of hospital management, which may have an impact on technical efficiency (25–27). The improvement of the performance of public hospitals requires an understanding of the factors and components of technical efficiency, as well as the cooperation of health administration, policy planning and daily operation management (27). Consequently, this qualitative investigation was conducted with key health system stakeholders to better understand the reasons for inefficiency in public hospitals in KSA and explore potential mechanisms for improving hospital performance (28–30). This study builds on our previous work (24, 25) to fill gaps in available information to strengthen the validity of findings of quantitative assessments, contribute to feasible recommendations for developing healthcare policies for reaching efficient hospital services, and potentially support further efficiency studies and periodical monitoring of quality improvement and performance of healthcare services (24, 25).

The aim of this study was to explore the factors that influence the technical efficiency of public hospitals and the mechanisms behind the production process in KSA. To achieve this, we investigated the following specific objectives: (1) identify the factors that affect hospital efficiency from health systems stakeholders' perspectives; (2) illustrate the desired mix of inputs and outputs and service utilization in public hospitals, and (3) propose recommendations to enhance the technical efficiency and quality of health services in the public hospitals.

## Methods

### Study Design

The study employed a qualitative methodology, interviewing the key informants (KIs) using semi-structured interviews (SSIs) as the data collection tool. SSIs were conducted with health system stakeholders drawn from public health facilities and the MOH. The SSIs were designed to identify key barriers to hospital efficiency and specific factors that influence efficiency and performance in respect to the use of inputs, outputs and production processes. We also sought suggestions for improving efficiency in public hospitals in the KSA.

### Study Participants

The study participants were drawn from the three levels of the health system: national (MOH), district and hospital. They were current and former senior officials, health administrators and hospital managers. We focused mainly on the views of these stakeholders at management and ministerial levels because they were the main decision-makers regarding performance and efficiency at the hospital level and deemed by the research team to be appropriate for providing useful insights into the study topic.

### Participant Recruitment

Participants were selected using purposive and snowball sampling techniques (28). They were identified in consultation with local academics colleagues, who had professional experience in public hospitals and familiar with the organizational structure of the MOH, and constituted as an advisory team to the study. Participants were identified based on their role in the MOH and potential to provide meaningful information to help in addressing the study objectives (29, 30). During the recruitment process, we reviewed the organizational chart of the MOH to identify relevant individuals, with particular emphasis given to individuals who held cross-sectoral roles and involved in organizational strategy, operations and performance. Initially, selected officials were reviewed by the local academic colleagues, with the final list of potential participants achieved by consensus among the research team members (30). All potential participants were contacted and invited to the study, with follow up emails/calls made if they did not respond to the initial invitation (28); an appropriate time and place for the interview were agreed with those willing to participate. Additionally, we asked interviewees to recommend other experts within the field so that they could be interviewed; this yielded useful informants who were included in the study and enabled a wide range of views on the topic to be captured (31). A total of 34 potential participants were identified and invited to the study; 20 of these accepted to participate (Table 1), with time constraints given as the reason for non-participation. All the participants were male as women did not work in that types of positions of the target informants.

**Table 1:** Characteristics of the study's participants (*N* = 20).

**Participants *n* = 20**	***N* (%)**
**Sex (Male)**	20 (100%)
**Age (years)**	
25–35	1 (5%)
36–45	11 (55%)
46–55	6 (30%)
>55	2 (10%)
**Official position**	
MOH officials	4 (20%)
District health administrators	5 (25%)
Hospital managers	11 (55%)
**Experience (years)**	
2–5	3 (15%)
5–10	8 (40%)
10–15	6 (30%)
>15	3 (15%)

### Data Collection

Data for the study were collected between July and September 2019 using semi-structured interviews (SSIs). The interviews were conducted by the principal investigator (PI) through face-to-face contact or Skype call. Topic guides were developed for the interviews and were informed by the literature on hospital efficiency and findings from a previous quantitative study conducted in the study context by the PI. The guides covered a wide range of topics on efficiency, including the concept of technical efficiency, factors affecting efficiency in hospitals, resources for health services delivery (inputs and outputs); mechanisms for hospital performance; and recommendations for improving hospital efficiency (Supplementary Material 1). Each interview lasted for approximately 1 h. They were recorded digitally with permission from participants and complemented with handwritten notes. All interviews were conducted in Arabic as it was the first language of both the PI and the participants and later translated into English to facilitate analysis. During the interviews, the PI ensured that participants understood the interview questions, including the concept of technical efficiency, before their responses were elicited. Data saturation was reached with 20 interviews as no new themes emerged at daily debriefing meetings with the research teams. Hence, no new participants were identified for further exploration on the topic.

### Data Management and Analysis

Audio recordings were first transcribed verbatim in Arabic and given back to the interviewees for verification. These were then translated into English by the PI, with the quality assurance provided by a local professional translator (28). Consistent with the General Data Protection Regulation (GDPR) guidelines for data security (32), participants names and other identifying information were anonymised, and the data saved in a password-protected Excel spreadsheet to enhance data confidentiality. Data were analyzed in QSR NVivo 12 software based on a thematic analytic approach, noted to be appropriate for interdisciplinary and collaborative studies (33). A coding framework was developed based on emerging ideas from the data as well as the topic guide and the study objectives. The framework was developed with flexibility to accommodate emergent new themes as the coding evolved. Using the coding framework, each transcript was read for recurrent ideas and assigned relevant codes. Similar codes were grouped to form themes that were used to address the study objectives. The emerging themes were discussed during regular meetings with research team members (31).

### Ethical Consideration

Ethical approval was obtained from the Ethics Committee of Institutional Review Board of the King Fahad Medical City, the Ministry of Health in Saudi Arabia (IRB log No. 18-166E), and the Research Ethics Committee of the Liverpool School of Tropical Medicine (Ref: 19-036) (Supplementary Material 2). All participants were furnished with information about the purpose, risks, benefits and procedures of the study and written informed consent was obtained before data collection (Supplementary Material 3).

### Patient and Public Involvement

Neither patients nor the general public was involved in the design, conduct, reporting or dissemination of our research.

## Results

Key findings from the data are presented in three broad themes based on the study's objectives: (1) components of public hospital performance in respect of health services (outputs) and health resources (inputs) and community health demands; (2) factors influencing the efficiency and utilization of health resources; and (3) recommendations for improving hospital efficiency.

### Performance Components for Public Hospitals

#### Health Services Provision

The majority of key informants (KIs) and health care providers noted that inpatient and outpatient services were the most frequent health services provided by public hospitals (Table 2). Emergency services were commonly demanded from the hospitals. Also, most of the KIs noted that several surgical services were provided to support health outcomes in hospitals. Supportive therapeutic and diagnostic medical services were also identified by almost all participants as important in health services delivery in the hospitals. Many KIs indicated that preventive procedures are fundamental to improving health outcomes.

**Table 2:** Components of performance in the public hospitals.

**Category**	**Sub-themes**	**Quotations**
1. Health services	Outpatient services	“*The hospital ensures the provision of diagnostic and therapeutic services to all patients”* [Hospital Manager (HM)].
	Inpatient services	“*Inpatient services are mainly provided by secondary care hospitals”* [District health administrator (DHA)].
	Emergency services	“…especially the treatment of accidents and fractures” (HM).
	Surgical services	“*Surgical therapeutic services, including cardiology, neurosurgery, obstetrics & gynecology and general surgery, are main surgery services provided by the hospitals”* (HM).
	Supportive therapeutic & diagnostic services	“*Support Services, e.g., laboratory, pharmacy and nutrition, are necessary services required to produce health outcomes in the hospitals”* (MOH official).
	Preventive Services	“*Preventive Services, like, vaccinations, health awareness and infection control are essential to improve the public health”* (HM & a DHA).
2. Services demanded by communities	Emergency, outpatient & pharmacy services	“*The most demanded health services are emergency, outpatient and pharmacy services, especially in the hospitals located in a place near to the pilgrim's or international roads”* (DHA).
3. Health resources	Health workers	“*Health workers, across the different health specialities, are the backbone of hospitals as health service provisions depend mainly on the clinical staff”* (HM).
	Capital resources	“*The clinical capacity of the hospital and (medical and non-medical) devices are the essential resources in the public hospitals”* (MOH Official).
	Purchase budget	“*Funds for the purchasing supplies and medicines should be done by the hospital itself, to reduce purchasing time”* (HM).

The KIs justified the selection of the services based on three reasons: First, the MOH health strategic plans, health management and the hospital objectives. A noted: “*The selection of services is made based on the health plans, the scale of service and the clinical capacity of hospitals in line with the objectives of the MOH and the hospital's”*. Second, based on hospital speciality, a HM said: “*This classification is considering the scope of service and speciality of hospitals*.” Third, based on community demands for specific services, a DHA stated: “*the needs from the community in the area and its nearby environment, which led to establishing the service scope of the facility.”*

#### Health Services Demanded by the Communities

The KIs identified emergency, outpatient and pharmacy services as the most commonly demanded health services by the communities (Table 2). They noted that the demand for health services in hospitals could be influenced according to socio-economic characteristics of the patients. A HM said: “*The elderly and low-income people have a greater need for general health services such as emergency, outpatient and pharmacy services as compared to younger and higher-income people who can benefit from the private sector*.”

#### Resources Utilization in Hospitals

All key informants mentioned that the contracted health workers, capital resources and infrastructure are essential resources to deliver health services in the hospitals. Many KIs identified that the periodically consumable supplies, such as medicines and dressing materials, are essential to providing services for patients. They also suggested allocating specific budget in each hospital for purchasing these supplies without delay (Table 2).

### Barriers to Efficiency and Utilization of Health Resources in Public Hospitals

**Figure 1:**
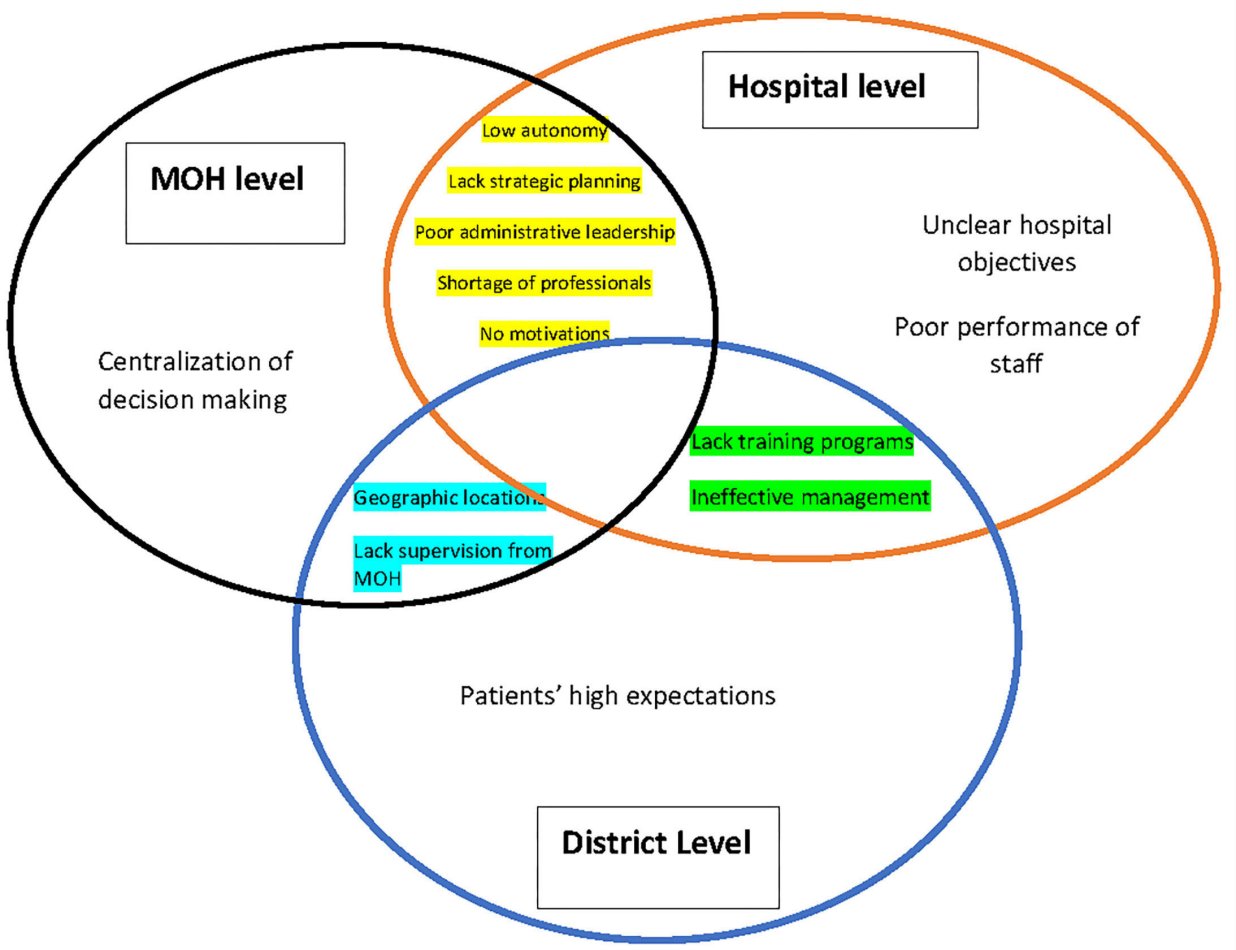
Summery of barriers of efficiency and utilization of health resources in public hospitals.

The KIs identified the barriers that affect the efficiency and utilization of health resources in public hospitals (Figure 1).

#### Ineffective Hospital Management

The KIs identified the ineffective management of health resources and weak administrative leadership, and lack of competent hospital managers are the main challenges of efficiency in public hospitals. A DHA said: “*There have been observed poor administrative and leadership performance among some hospital leaders. These health leaders lack the required ability to utilize existing resources to meet the requirements of health services*.” Several KIs indicated that the lack of clear objectives and goals and lack of advanced strategic planning as major obstacles to the performance. A DHA said: “*There is a blurred vision of health managements and lack of clarity of the aim of hospitals to make the best use of medical specialities and health resources*.”

#### Lack of Monitoring

Many KIs indicated that poor supervision, lack of annual assessment of staff performance and the follow up led to the failure to meet the standards in health service provision in many hospitals. Some KIs also mentioned the lack of identification of causes of resource wastage and the appropriate application of policies and procedures as barriers to good performance. A MOH official stated: “*The standards of health service providers are not followed, which exacerbate critical health conditions and reduce quality and safety and have a huge impact on medical intervention. There is a lack of supervision and follow-up from the MOH*.”

#### Centralization of the Decision Making

Many KIs claimed that centralization of the decision-making at the MOH are major barriers to efficiency in public hospitals. A HM said: “*One of the major problems is the centralized decision-making for distribution of resources without referring back to the specific plans of hospital managements. It should be conducted according to needs for health services in the area*.” The lack of autonomy and power of hospital managers is another factor of inefficiency. A HM stated: “*Giving autonomy to hospital managers has a significant impact on hospital performance as a manager is the first leader of operational efficiency. Also, it is required to overcome routine obstacles that may be behind the failure to provide on-time health service. Nevertheless, there must be control and monitoring from the MOH affairs*.”

#### Availability of Skilled Health Workers

Some KIs have indicated that the poor performance of the medical practitioners and lack of relevant training programmes, which lead to poor medical outputs and poor quality of services. A DHA stated: “*There are poor performance and lack of experience and technical skills of health practitioners, administrators, and hospital management to provide medical services. In addition, there is a lack of ongoing training programmes for health staff* .”

The shortage of professional medical staff was also a major barrier to hospital performance, especially those of rare specialities such as neurosurgery. Moreover, poor allocation of current specialists based on needs and demands of hospitals was raised as a concern. A HM stated: “*Lack of health professionals is a major issue in the hospital, where most of the specialists prefer higher wages and better work conditions, which often are available in private hospitals. Unfortunately, there is no attraction or encouragement for them in public hospitals; for example, there are no favorable promotions for their efforts. In addition, the public hospitals lack the optimal allocation of the existing specialists*.”

#### Accessibility to Hospital Services

The geographic location of hospitals plays an important role in accessibility to health service, according to many KIs. A HM stated: “*The geographical location of the hospital, if far from the patients and absence of transportations, are obstacles to hospital performance and the demand for health services. This problem also leads to worsening the health conditions for most patients in urgent need.”*

#### Population Demands

The demand for health services could be changed based on different seasons in the year and the type of health service. For instance, during school time of the year, pediatrics and vaccination health services were most demanded compared with other services. A HM said: “*The patient's demand for hospital services also vary across seasons of a year; for example, we face more demand for health services during winter compared to summer*.”

#### Patients' High Expectations

Many KIs argued that patients' high expectations is a serious challenge of hospital performance. A DHA said: “*We face challenges of hospital performance as the patients have high expectations in respect to the services they will receive. For instance, lack of understanding of the disease's nature and awareness about suitable health clinics lead to a long waiting time. Eventually, patients will lose confidence in hospital services*.”

### Feasible Options to Improve the Efficiency of Public Hospitals

The study participants discussed the potential changes needed in the current health system to improve the efficiency of public hospitals in Table 3.

**Table 3:** Feasible options to improve the efficiency in the public hospitals.

**Sub-themes**	**Quotations**
Hospital objectives	“*The hospitals need to adopt MOH aims in developing strategic plans in line with the capabilities, logical ambition and the needs of the patients and the community of that hospital*” (MOH official).
Hospital management	“*We need to establish specific criteria and set standards by the health committees to select hospital leaders at the level of the MOH and each district for deploying competent hospital managers”* (MOH official).
Decentralization of powers	“*To improve the performance, we need decentralization of hospital management and involve health workers in decision-making”* (HM).
Efficiency assessments	“*There must be an analytical study of the causes of the waste in hospital resources and find practical solutions to treat it and generalize the successful experiences”* (MOH official).
Performance measurements	“*We need to develop a performance measurement system under the supervision of MOH to assess the health resources used to produce the services as well as considering the patients' feedback on their consumed services”* (DHA).
Research and education	“*It is essential to educate and raise awareness of health practitioners about the importance of operational efficiency of hospitals and value of health services*” (DHA).
Health practitioners	“*The MOH should consider activating the part-time contracts (e.g., LOCUM) to overcome the need for specialized medical services (e.g., neurosurgery). It is also needed to establish a system of promotions for health workers to retain them in the hospital*” (HM).
Health education	“*I encourage to promote health education in the communities to raise patients' awareness about health services they receive and gain the patient trust in the hospitals*” (HM).
Health information	“*We need to establish health reporting systems focusing on patient's information, medical history and clinical cases, linking electronically with other departments, hospitals and the MOH. This database also should include health resources and services provided*” (HM).

#### Hospital Objectives and Strategic Plans

The hospital objectives need to be set in the light of the MOH aims and develop the relevant strategic plans through the teamwork of the hospital's staff in order to specify the clear goals and identify the clinical tasks for improving health outcomes and specify the job description of each practitioner.

#### Criteria for Hospital Management Staff

Most of the participants noted the importance of developing an explicit criterion for the selection of hospital managers and link their performance to MOH plans and hospital objectives through periodical meetings and direct supervision by the MOH. It is also important to start a training programme for all health leaders focusing on the practical steps to achieving the objectives of the hospital and improving the performance.

#### Decentralization of Powers

Many KIs have emphasized the need for decentralization of decision-making and securing more autonomy of hospital managers in the reallocation of health resources to improve hospital performance. For instance, enhance the authority of hospital managers to facilitate the provision of medical staff, equipment and consumables at no time.

#### Efficiency Assessments and Resource Allocation

Almost all the KIs recommended the need to investigate the wasting of resources in some hospitals, also to conduct studies that compare the efficiency between the hospitals to learn lessons and improve the quality, practice and management of health services (Table 3). They also supported the need to reallocate health resources from inefficient hospitals to those with higher efficiency through regular meetings with the health affairs of the districts and based on the findings of efficiency analysis and community demands.

#### Performance Measurements

There is a need for applying policy and procedures to assess the performance in the hospitals, like through mandatory monitoring programmes by the MOH. Such actions should include hospital performance through patterns and the quality of services delivered. It should additionally include the feedback of patient's experiences and field visits by the health affairs in the region.

#### Investing in Research and Education for Improving Efficiency

Several KIs raised the importance of education on efficiency among health workers and leaders, application of further efficiency research on public hospitals, and learn from the experiences of other hospitals to guide the policy implications. They put emphasis on developing training programmes about the effective utilization of health resources among health workers. Also, it is important to apply the implications of efficiency research findings and link to improvement not only at the operational level but also at the level of leadership development.

#### The Working Environment for Health Practitioners

The KIs demonstrated the necessity for solving the shortage of specialized staff in public hospitals and they also highlighted the need for improving the attraction factors for the current health workers. They suggested the need to activate part-time recruitment of specialized practitioners and develop measures to attract such professionals, like performance-based promotion as incentives and promotions (Table 3). They also highlighted the need for training programmes on skill development and performance improvement of staff, as well as resource optimisation and management.

#### Health Education in the Communities

The majority of the KIs explained the importance of supporting the awareness about health services through health education in the communities, understanding the patients' health needs and improvement of the scope of service and demand for health in the hospitals. Such awareness can be reached through community participation in periodical meetings with health affairs, also through media and social networks.

#### Strengthening Health Information Management

The KIs discussed the poor availability of patient records, resources consumed and services delivered, which and which have negatively affected the performance assessments and any future planning. The need for establishing an integrated recording and management system of all patients data and services provided, which were linked with different hospitals and specialities and monitored directly by the MOH. This required training of clinical staff and allied health professionals on data recording services and the resources used.

## Discussion

This study examined the views of a range of health system stakeholders on the barriers to efficiency in public hospitals in KSA and measures to mitigate these. We reviewed components and mechanism of performance in the public hospitals, factors that affect the efficiency, barriers, and remedial actions from the viewpoints of MOH decision-makers and health professionals in the KSA.

The findings highlighted the essential health services that public hospitals should deliver to the population, which have a fundamental role in hospital performance and provided in public sector secondary care facilities and tertiary level hospitals worldwide (6, 14, 34). In the KSA, the services were chosen for three reasons: the strategic plans of the MOH, the objectives of the hospitals and its scope of services, and the community demand for these health services. Emergency care, outpatient care and pharmacy services were mainly demanded by the communities in the catchment areas of the hospitals, which was observed in other studies in Saudi Arabia (35, 36). This demand for these services is due to easy access and availability in public hospitals. In addition to, poor performance and quality of health services in other health providers in the hospital's area, i.e., primary care services, which led patients to seek for these services in the hospitals as observed in the literatures (37). We suggest enhancing the incorporation of primary care centers in service provisioning beside the hospitals through developing the referral policy and better coordination among primary care centers, hospital administration and the MOH (36).

The community demand for health services can be changed according to the demographic and socio-economic characteristics of the populations. For instance, the utilization of health services in public hospitals was higher among the elderly, low-income and less-educated populations, according to the KIs. Similarly, several previous empirical researches argued that more impoverished and older people utilized more services due to their free access to public hospitals (38, 39). In addition, previous quantitative research in KSA found that socio-economic characteristic of the population (like low-income people) had a significant impact on hospital efficiency (25). The scope of health services may have to consider the characteristic variations of the catchment population in amending healthcare policies. The health resources (inputs) that mainly were utilized in public hospitals were in the same line of some other research findings from the public hospital performance studies in KSA and worldwide (7, 15, 24).

KIs identified the efficiency challenges that were faced by public hospitals and proposed potential solutions to overcome those barriers. Ineffective administrative leadership and poor management of the public hospitals appeared to have a major impact on efficiency. The KIs, therefore, suggested the urgent need for developing selection criteria of hospital managers and health leaders, paying attention to the qualifications and work experiences of the managers since their roles seem to be the key to the hospital performance. We recommend direct supervision of performance of the managers by the Health Affairs and the MOH. A similar study conducted by Perera and his colleagues in 2000 also found that the hospital management, and the characteristics of the managers, including qualifications and administrative experiences, could influence hospital efficiency (40).

The participants discussed the impact of centralized decision-making and lack of autonomy of hospital managers on hospital efficiency. This discussion was supported by the previous literature, which argued that more decentralization and autonomy of the managers and flexibility over the management of health resources, procurement and services delivery process in response to people's need would have a significant impact on hospital performance and efficiency (11, 41, 42).

In addition, the unclear objectives of hospitals and lack of advanced strategic planning in service delivery and insufficient supervision by the MOH were essential barriers to hospital efficiency. Therefore, it can be suggested that the hospital administrators and staff should have a clear understanding of the hospital goals, relevant strategic plans and specification of the role of each health worker following the aims of the MOH as well as the need for health services of the community. Previous literatures revealed the link between explicit strategic health plans and their positive impact on the competition of hospital production and efficiency in public hospitals (28, 43).

The poor performance of medical staff and lack of training programs on effective utilization of health resources were indicated as the critical determinants of inefficiency as the health practitioners are the cornerstone of hospital performance and direct provider of health service. For improving efficiency, it is thus important to develop the training programmes and monitor the hospital staff for improving the performance of health practitioners and quality of services they deliver by, for instance, applying policy and procedures of key performance indicators (KPI) for health practitioners in each hospital. Performance monitoring, training programmes, and reward and recognition of staff have a significant impact on operational efficiency, which are commonly found in the literature on hospital performance (44–46).

The shortage of professional staff, particularly in rare specialities, and the lack of motivation, as well as the retention of current healthcare workers in public hospitals, were significant barriers to hospital performance toward efficiency (44). The challenge of the healthcare professionals retention was due to the absence of the factors of attractions to retain them in public hospitals, for example, low salaries and poor working conditions. In competition with other providers (like private facilities), which offer better benefit packages for recruitment, public hospitals become unattractive for healthcare professionals. The factors of attraction for retention of healthcare professionals, which have a substantial impact on the performance, were discussed in the literature worldwide and in the Saudi context (46–48). Following the recommendations by KIs and findings in the literature, we focus on improving the work conditions, including promotions and encouragements for healthcare workers, on retaining them in public hospitals. Additionally, the scope of recruiting part-time staff of rare services, for instance, LOCUM contracts, should be useful in this context.

The patients' high expectations of healthcare services and changing the demands for services and geographic location of the hospitals have affected efficiency. Several studies argued that the high expectations of the patients regarding healthcare services and other related experiences had a significant effect on the demand for healthcare and consequently the delivery of healthcare services in hospitals (45, 49). It is, thus, required to expand the awareness of healthcare service levels in communities through public health education events and social media. Also, policymakers may pay attention to understanding the healthcare needs among different populations and review the scope of services in public hospitals accordingly (50).

There is a growing need for education in the operational efficiency concepts and training programmes among healthcare workers, which aims to improve the effective utilization of healthcare resources and enhance service quality and production, as well as the improvement of the value for money in hospitals. It is also important to conduct further technical and allocative efficiency assessments for public hospitals, providing an exchange of knowledge from such assessments with healthcare providers and public hospitals. The research-based learning of successful experiences needs to be disseminated for policy implications and future strategic plans. Previous investigations observed that training healthcare workers would improve their performance and productivity, which should be included in the hospital's policies to have a significant impact on the efficiency of public hospitals (51, 52).

We emphasized the poor statistics of health information regarding patient status, service delivery and quality of care in the hospitals, which made it difficult to assess the performance of these hospitals. This finding was supported by an investigation by Hollinworth (12). It was also noted that there was a continuing need for establishing an integrated recording system of all healthcare providers and facilities linked with each other under the supervision of health affairs in MOH. The integrated reporting system should contain patient's information, medical history and clinical cases, services provided (patterns and quality) and health resources used, as well as the treatment procedure. According to a broad range of scientific publications, a developed and integrated reporting system in healthcare was useful to understand the production mechanisms, extracting knowledge from previous mistakes to prevent them from happening thereafter. Such an integrated reporting system also contributed to the improvement of patient safety and effective utilization of healthcare services in public hospitals (12, 53, 54).

The efficiency analysis of the public hospitals has become an urgent demand and should be required by the MOH in the KSA, according to the KIs. We, therefore, encourage MOH to implement the policy and procedures in the hospital's performance assessments and regular monitoring of the hospital's service delivery as well as follow-up of the utilization of health resources in the light of strategic objectives of the healthcare plans. For instance, appropriate actions should be taken to measure the performance of each hospital based on the quantitative and qualitative evaluation of health services, considering the patients' feedback and the utilization of health resources. It, thus, will help identify the weaknesses in performance and find the best mix of health resources, which may contribute to reform evidence-based policymaking through a specific action plan for the given hospitals. Such directives were observed in the previous literature in their findings and suggestions (10, 55, 56).

The health administrators in MOH are required to understand the findings of efficiency analysis and the reasons for inefficiency in some hospital and compare with better-performed ones and relevant factors. It appeared to be important to evaluate the underlying factors (inputs and outputs) that affect the performance (both internal and external) of the hospitals so that lessons can be taken for future benefits from the mistakes and also from the successful experiences to improve the scientific knowledge of hospital efficiency. We, thus, encourage the application of scientific findings of the reallocation of health resources from lower to higher efficiency. In this context, we give emphasis on the manager's autonomy in the process of redistribution of resources. It should be noted here that similar findings and recommendations were observed in the various scientific literature (15, 24, 25, 57, 58).

### Study Limitations

The small sample size (20 senior health professionals) might be a limitation for the generalizability of the findings. To mitigate this limitation, we employed purposive and snowball sampling techniques which enabled us to identify participants who, through their positions, were able to contribute meaningfully to address the study objectives. Also, drawing the participants from different levels of the health systems, which enabled us to capture a wide range of views and triangulate in order to provide richness and reliability to the data, and to provide an in-depth understanding of the factors of inefficiency to create feasible recommendations based on real-life experience. We, further, kept the saturation of information under consideration in our analyses as 70–95% of the KI's answers were agreed, which we found adequately addressed.

## Conclusions and Recommendations

This qualitative study, involving policymakers and hospital managers at different levels, explored the existence and underlying factors of inefficiency in the public hospitals in the KSA and their potential remedies. The sources of inefficiency comprised the characteristics of health systems and hospitals as well as of the community in their catchment areas. Ineffective hospital management, lack of strategic planning and goals, weak administrative leadership, and absence of monitoring the hospital performance appeared to have a potential impact on hospital efficiency. The conditions of healthcare staff in respect to both skills, authority and psychological factors were considered to influence the efficiency level. Further, lack of appropriate data for decision making due to the absence of an appropriate health informatics system was regarded as a factor of inefficiency. At the community level, the insufficient information about the need for healthcare and expectations of the patients were considered as a hindrance to adopt and/or reform health policies to be efficient in the provision of services.

As remedies, considerations to the aims of the hospital and health system as well as socio-economic and demographic characteristics of the catchment areas were recommended to adopt by the hospital administration. Simultaneously, empowerment of the hospitals for decision-making, skill development of managers, equipping with clinical staff for critical healthcare and monitoring of performance were recommended. Some of the remedies can demand more resource involvement (like for monitoring, supervision, training), but such actions are expected to improve the hospital outcomes by using possible economies of scale and consequently may contribute to higher efficiency if not in the short-run but in the long run.

Based on our current investigation, we recommend enhancing more awareness about hospital efficiency and effective resource utilization for performing appropriate tasks among the stakeholders in the health system of the KSA. Due to limited empirical investigations of hospital efficiency in the country, more studies in this area will be useful for further verification of the findings of this research and for exploring new knowledge related to hospital efficiency.

## Data Availability Statement

The raw data supporting the conclusions of this article will be made available by the authors, without unduereservation.

## Ethics Statement

The studies involving human participants were reviewed and approved by Research Ethics Committee (REC) at Liverpool School of Tropical Medicine LSTM (Ref: 19-036; dated: 18/06/2019). The patients/participants provided their written informed consent to participate in this study.

## Author Contributions

AA, LN, MB, and JK contributed to conceptualizing the research question, study design, and settings. AA, MB, and JK reviewed the MOH structure charts for selection the proposed participants and revised the interviewes questions and plan the data collection process. AA conducted the interviewes in the KSA. AA and JK conducted the translation of the answers transcripts. AA and YA conducted the data analysis, interpretation, and writing of the manuscript. AA, LN, MB, YA, and JK contributed to writing, reviewing, and revising the manuscript. All authors finally reviewed the manuscript critically and approved the final version for submission.

## Funding

This work was funded by the Deanship of Scientific Research at Jouf University under Grant No. DSR-2021-01-0301.

## Conflict of Interest

The authors declare that the research was conducted in the absence of any commercial or financial relationships that could be construed as a potential conflict of interest.

## Publisher's Note

All claims expressed in this article are solely those of the authors and do not necessarily represent those of their affiliated organizations, or those of the publisher, the editors and the reviewers. Any product that may be evaluated in this article, or claim that may be made by its manufacturer, is not guaranteed or endorsed by the publisher.
