# Supplementary material for: Factors Influencing the Efficiency of Public Hospitals in Saudi Arabia: A Qualitative Study Exploring Stakeholders' Perspectives and Suggestions for Improvement
Source: Front Public Health. 2022 Jun 16;10:922597. doi: 10.3389/fpubh.2022.922597 (PMC9243553; doi:10.3389/fpubh.2022.922597)
Supplement: Supplementary file 1 [file Data_Sheet_1.pdf]

Interview Questions Guide

Evaluation of Health Services Efficiency of Public Hospitals in Saudi Arabia

The purpose of today's interview is to explain the factors that affect the hospital efficiency, through identify desired inputs to and outcomes of public hospitals, from your point of view. A hospital that produces the maximum amount of outputs from a given inputs, or provides a given output with least quantities of inputs, can be recognised a technically efficient. Hospital outputs or health services that include; outpatient services that patients receiving from a hospital, discharge or inpatient services, emergency, surgical operations, pharmaceutical prescriptions, radiology and laboratory tests. Where inputs or health resources used in a hospital are; 1- capital, e.g. hospital capacity, number of beds, medical devices, 2- labour (health professionals), e.g. physicians, nurses, etc, and 3- consumable resources e.g. medications.

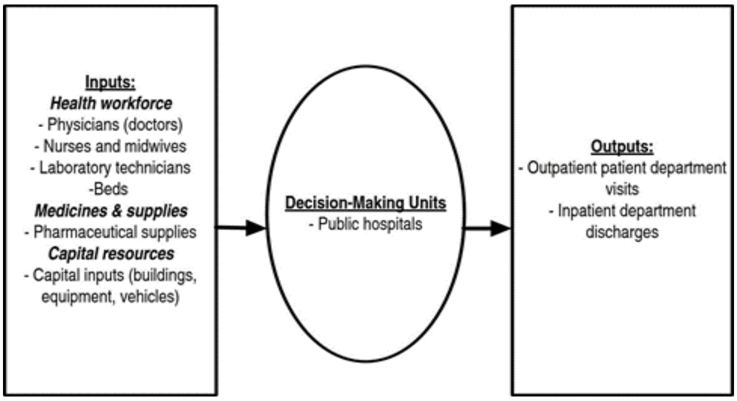

Your valuable information will be used for the making recommendations to reform the public hospitals towards improve the efficiency and performance.

Outputs (part one)

1. What are the outputs (health service categories) of the public hospitals delivers to the patients, please rank it in respect to their priority (Hospital goals)?
2. Why have you prioritized them this way? with respect to the objectives and outcomes of the public hospitals?
3. What health services do patients need and demand more than other services, and why?
4. Can the demand for specific services change according to the type of patients (average income for auditors, age groups, level of education, etc.)?
5. What are the main obstacles or difficulties that are opposed to providing adequate health services in public hospitals, if we assume that all health resources (clinical capacity, human forces, consumables) are sufficiently available in the hospital, and why?
6. Depending on your answer to the previous question, how can these difficulties be overcome? What is your practical advice that can be done in the hospitals?
7. What are the efforts that made by the Ministry of Health MOH to measure and monitor the efficiency of public hospitals? What is your advice in this regard?
8. How to improve the data reporting system to capture pattern and quality of service provisions in the hospitals?

Inputs (part two)

1. What are the main health resources (inputs) that the public hospitals use to produce the health services (outputs), rank please?
2. According to your answer to the first question, are there other health resources (other than you mentioned) that are critical to achieving the goals and vision of the hospital?
3. What are the barriers that affect the public hospital ability to transform these inputs into given outputs? (If we assume that a hospital has all required inputs)
4. What is the effect of hospital-managers autonomy on the efficiency of the given hospitals, and management on the production process? E.g. more autonomy will be helpful to improve a hospital performance or not. Why? Please provide recommendations.
5. How can the results of hospitals efficiency analysis be applied within the MOH system? Especially those that recommend reallocating resources according to the hospital's efficiency in providing health services. For example: If we assume that hospital A has low efficiency despite the availability of health resources, while hospital B is more efficient but suffers from some shortage of needed health resources. Can resources be reallocated from least (A) to highest (B) efficient in this regard? What are the practical steps to be followed?
6. In your opinion, what are the elements of measurement that must be followed in measuring health resources, services and the efficiency of public hospitals within MOH?

General statement (final)

1. What are the tips and recommendations you would like to provide to develop the efficiency of public hospitals in the Kingdom of Saudi Arabia? Do you have any final comments that you would like to make now when we are coming to the end of the interview?
