# Supplementary material for: Factors Influencing the Efficiency of Public Hospitals in Saudi Arabia: A Qualitative Study Exploring Stakeholders' Perspectives and Suggestions for Improvement
Source: Front Public Health. 2022 Jun 16;10:922597. doi: 10.3389/fpubh.2022.922597 (PMC9243553; doi:10.3389/fpubh.2022.922597)
Supplement: Supplementary file 3 [file Data_Sheet_3.PDF]

Letter of Information and Consent Statement

Evaluation of Health Service Efficiency of the Public Hospitals in Saudi Arabia

You are invited to participate in the research study titled "Evaluation of health service efficiency of the public hospitals in Saudi Arabia", led by Ahmed Alatawi, Dr. Jahangir Khan and Prof. Louis Niessen of the Liverpool School of Tropical Medicine in the United Kingdom. This consent statement explains the research study and its goals and objectives so that you will have the opportunity to discuss this and ask the questions you may have prior to your participation. If you agree to participate in this study, you will be requested to sign the consent statement at the end of this document.

Purpose of the Study

The aim of this study is to extract the factors that influence the hospital efficiency and the mechanisms behind the production process from the view points of the relevant stakeholders. For this purpose, we intend to explore the inputs, outputs and outcomes as well as their interactions for better understand the factors that affect the efficiency. This research will be useful for creating knowledge for a better measurement of the hospital efficiency. Subsequently, decision makers should be able to improve health policies to enhance efficiency and quality of public hospitals in Saudi Arabia.

Procedures of the Study

By signing this form, you agree to take part in-person or in a telephone interview. The interview should last approximately 45 minutes and will be scheduled at your convenience in desired time and place.

Confidentiality

All data collected during this study will be kept in secure papers in private place and electronic files that are accessible only to the principal investigator. Your name, location, contact information and all identifications will be kept separate from your interview transcript, which will be anonymous number/letter code. Your data (answers) will be analysed along with the data of several other participants. These will be described in the research reports, and some of the issues may be showed

with specific quotations from the interviews. If you are quoted in the research reports, your identity will be kept confidential so that readers cannot attribute the quote directly to you.

The Costs and Benefits of Taking Part in the Study

There are no physical risks involved in participating in this study. The only cost to you is the time you take to talk with the interviewer. To eliminate or decrease that cost, you can choose the time and location of the interview that are convenient to you. Also, you may withdraw at any time from the study without prejudice. There will be no direct benefit from participating in this research. However, we hope that the results of this study will help other researchers and policy-makers to develop tools for measuring efficiency of public hospitals in a better way. The health systems of Saudi Arabia should be benefited from this study.

Voluntarism

Your participation in this research study is completely voluntary. You are free to withdraw at any time from the study and without prejudice. If you withdraw during the interview, we will stop the interview and you will be asked whether you would like to have the data that you have provided to be used in the study or not. Also, you can specify any selected parts of the data to be destroyed, if you feel it revealed sensitive information.

Questions

If you have questions, or concerns or require more information about the study, please

contact below:

Ahmed Alatawi
Principal investigator
Health Economics research group
Department of Clinical Science
Liverpool School of Tropical Medicine
Email: ahmed.alatawi@lstmed.ac.uk
Phone: 001491496786

Signature of study participant

Evaluation of Health Services Efficiency of the Public Hospitals in Saudi Arabia

I have read and understand the preceding information thoroughly for the study. I have had the opportunity to ask questions, and all of my questions have been answered to my satisfaction. I agree to voluntarily participate in this study. I understand that I will receive a signed copy of this form. I hereby, declare that I have not been subjected to any form of coercion in giving this consent.

____

Name of the participant

____

Participant’s Signature   Date :

____

Name of Principal investigator

___________________________________   Date:

Signature
